# Supplementary figures and images for: Temporally integrated single cell RNA sequencing analysis of PBMC from experimental and natural primary human DENV-1 infections
Source: PLoS Pathog. 2021 Jan 29;17(1):e1009240. doi: 10.1371/journal.ppat.1009240 (PMC7875406; doi:10.1371/journal.ppat.1009240)

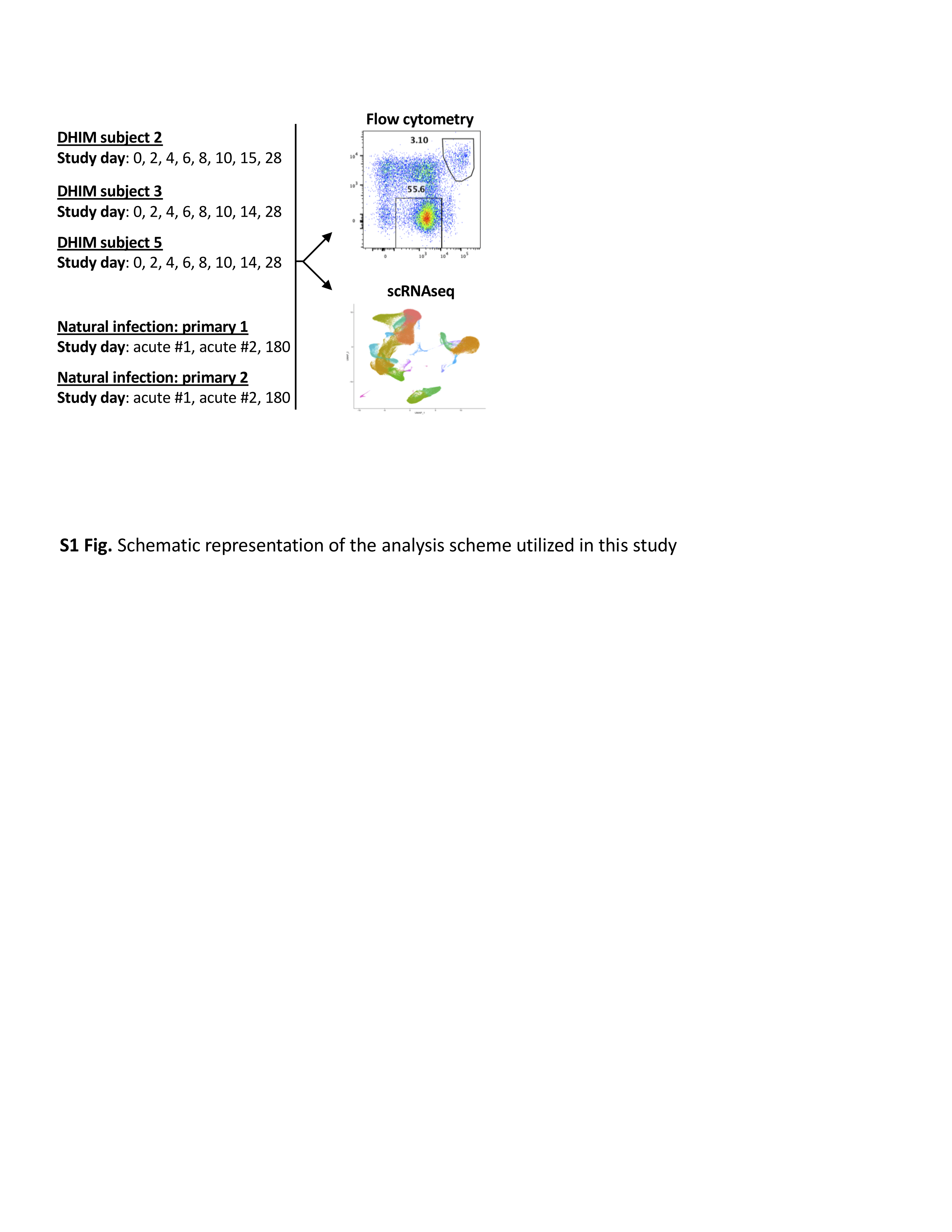

Supplement: S1 Fig — (TIF) [file ppat.1009240.s001.tif]

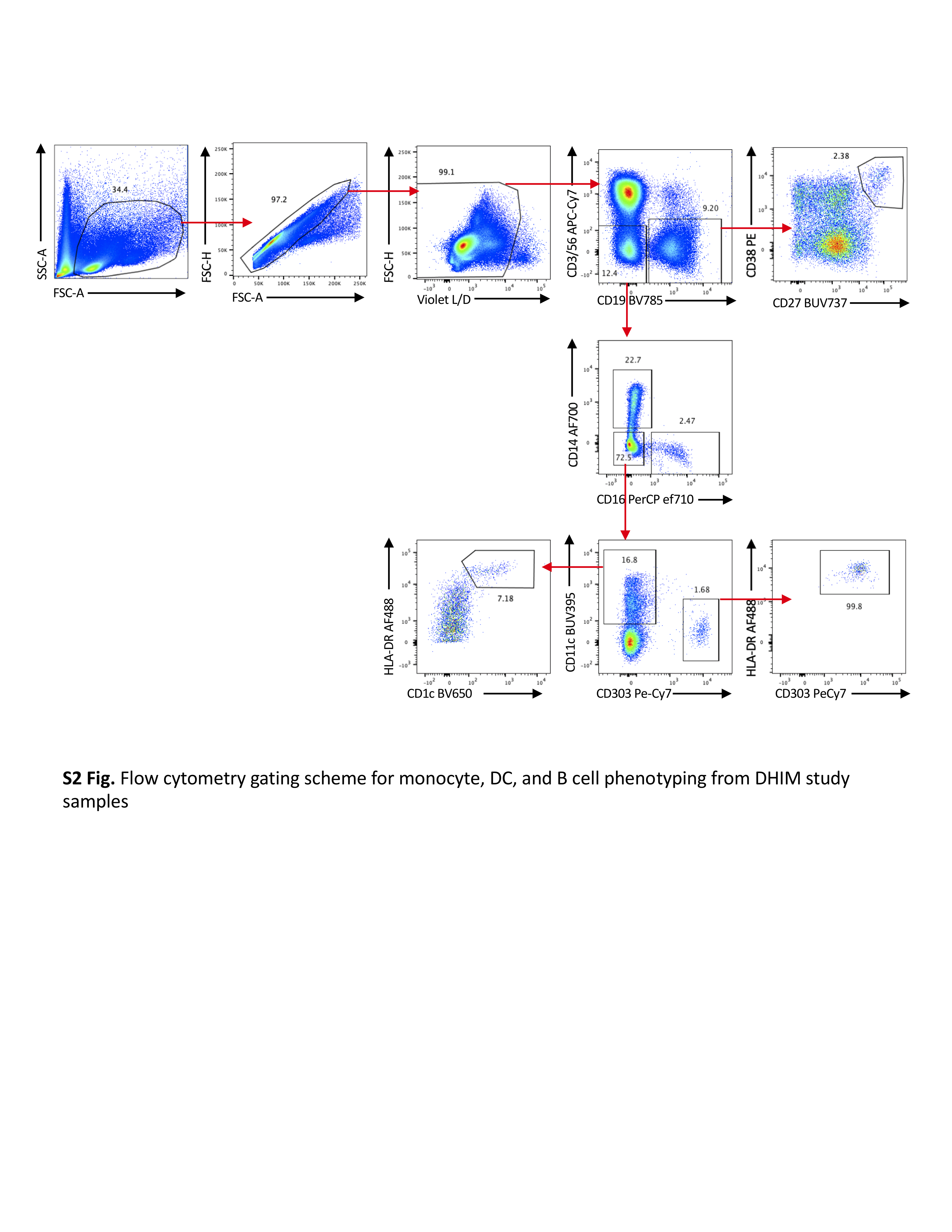

Supplement: S2 Fig — (TIF) [file ppat.1009240.s002.tif]

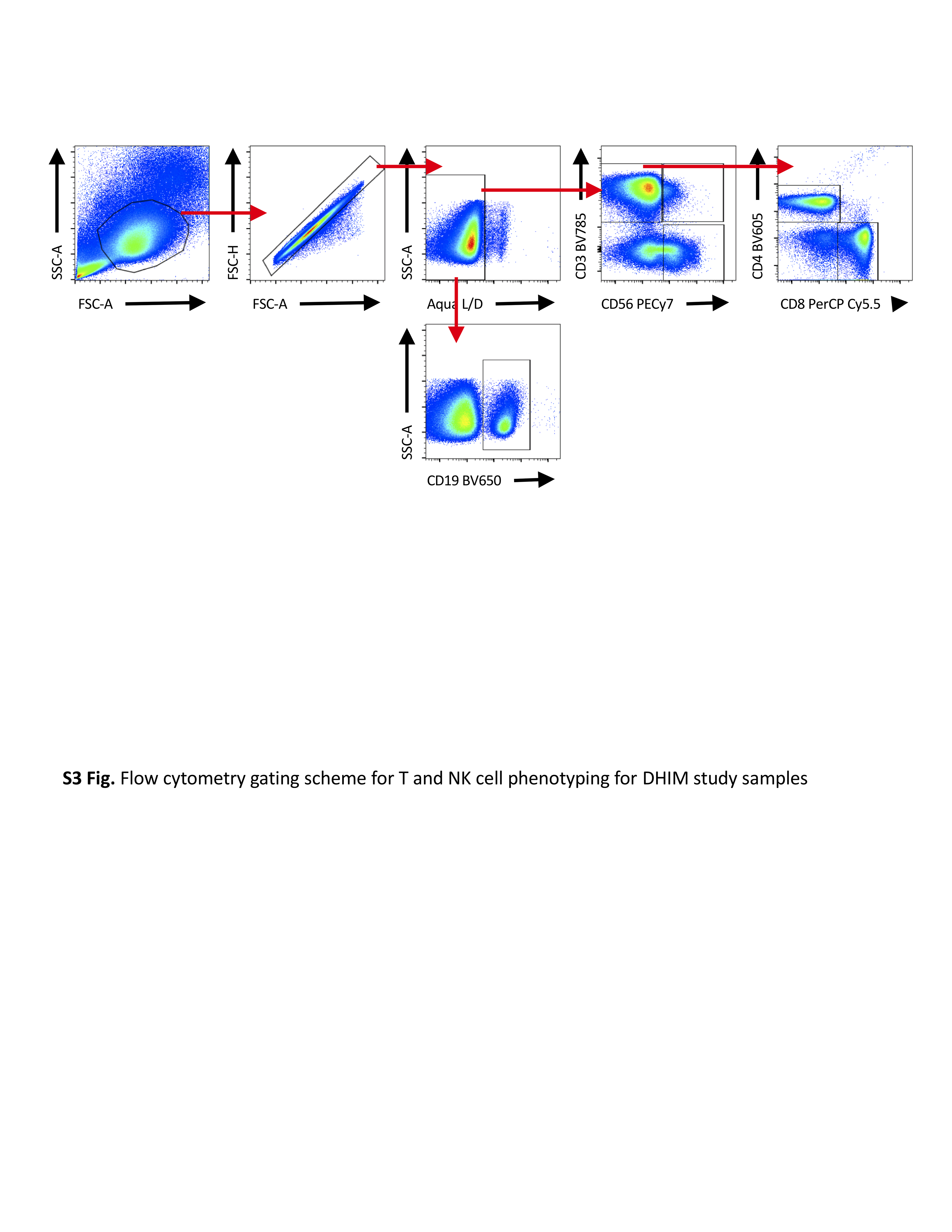

Supplement: S3 Fig — (TIF) [file ppat.1009240.s003.tif]

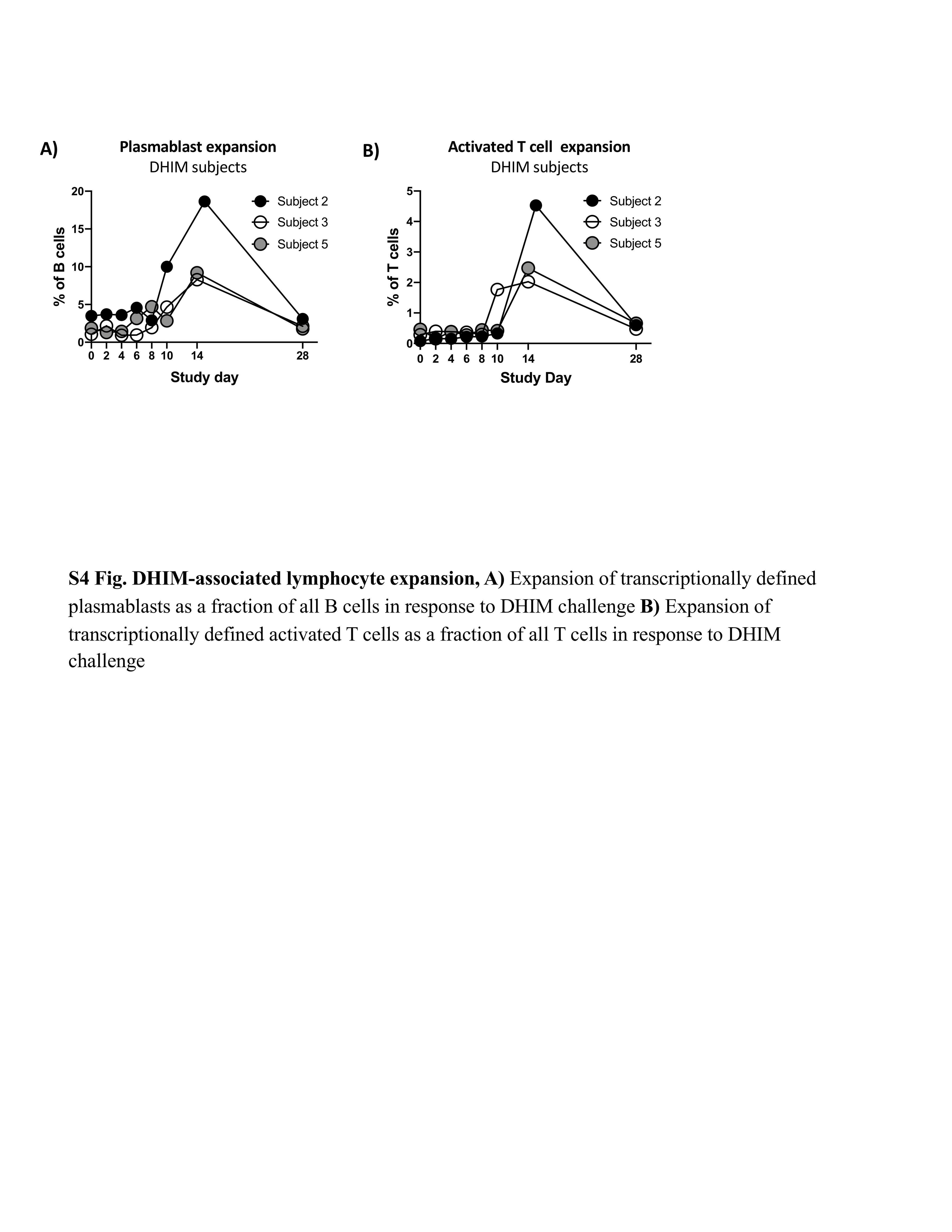

Supplement: S4 Fig — Experimental primary DENV-1 associated lymphocyte expansion, A) Expansion of transcriptionally defined plasmablasts as a fraction of all B cells in response to experimental primary DENV-1 infection B) Expansion of transcriptionally defined activated T cells as a fraction of all T cells in response to experimental primary DENV-1 infection. (TIF) [file ppat.1009240.s004.tif]

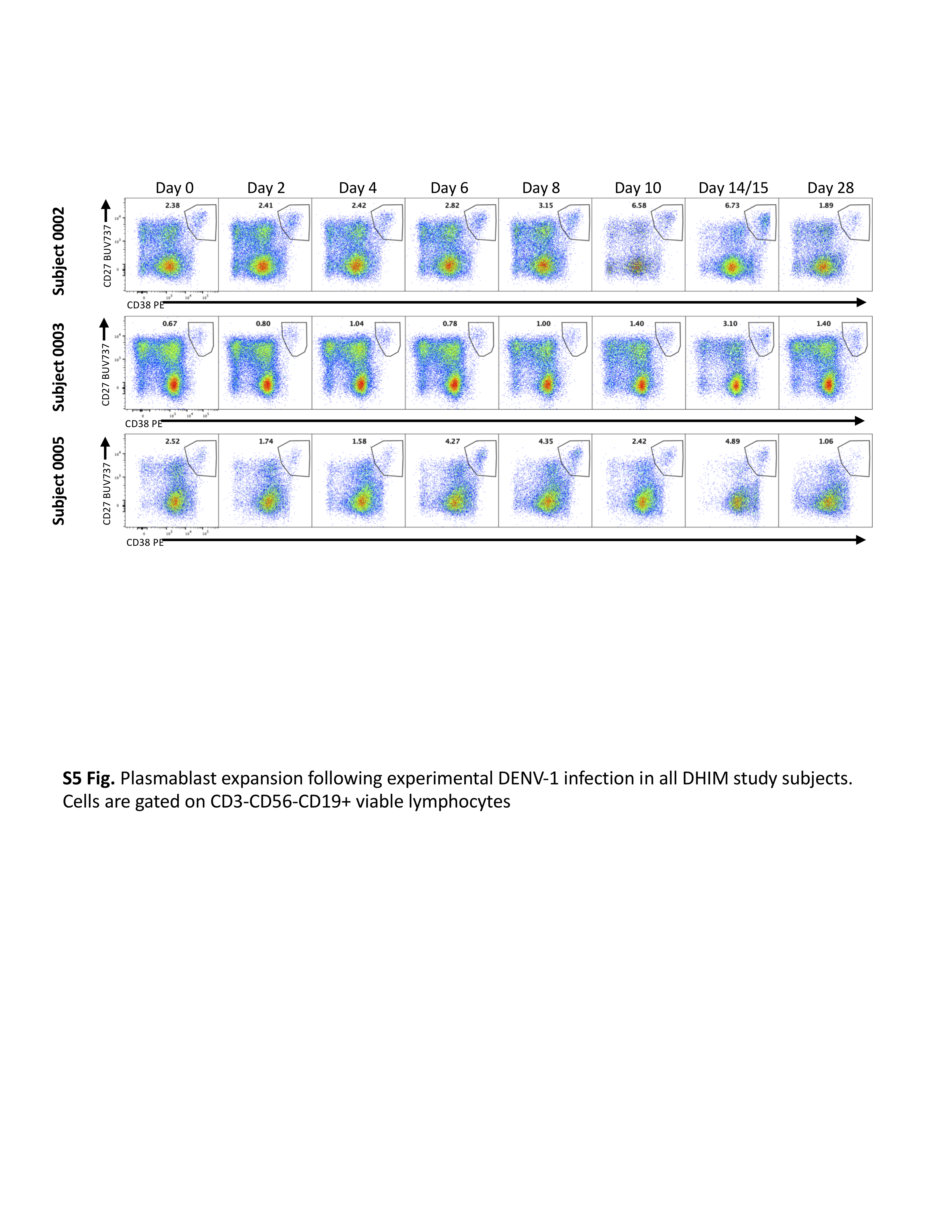

Supplement: S5 Fig — Cells are gated on CD3-CD56-CD19+ viable lymphocytes. (TIF) [file ppat.1009240.s005.tif]

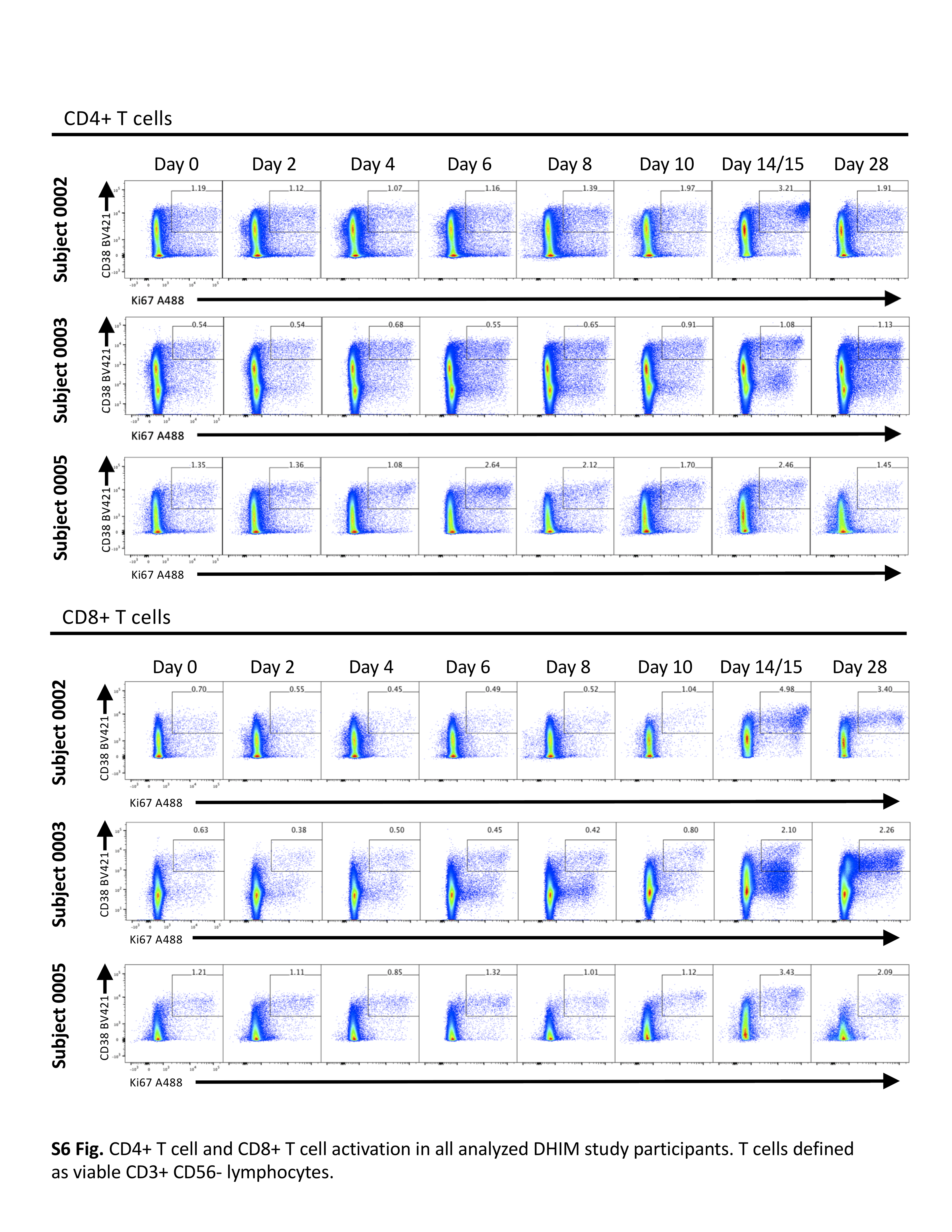

Supplement: S6 Fig — T cells defined as viable CD3+ CD56- lymphocytes. (TIF) [file ppat.1009240.s006.tif]

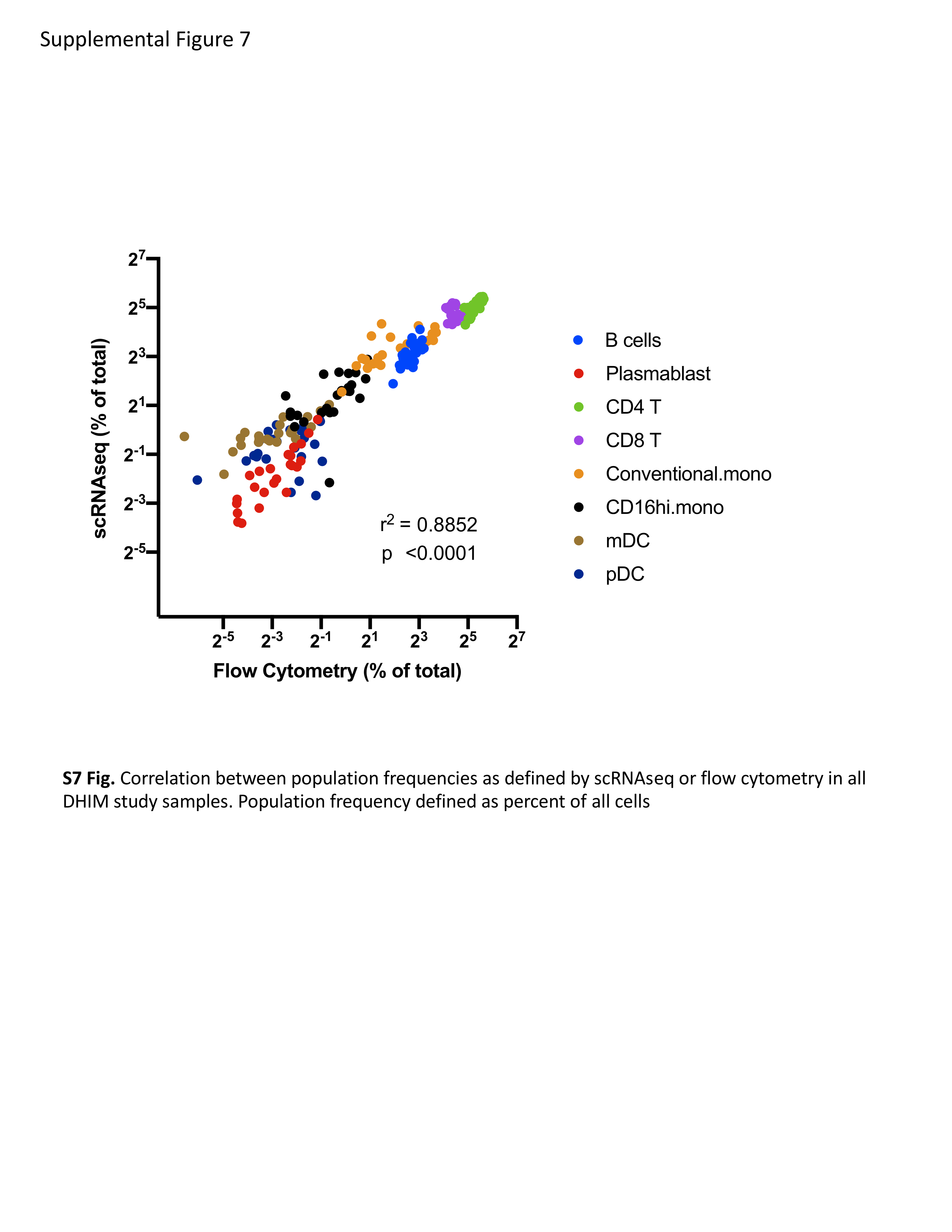

Supplement: S7 Fig — Population frequency defined as percent of all cells. (TIF) [file ppat.1009240.s007.tif]

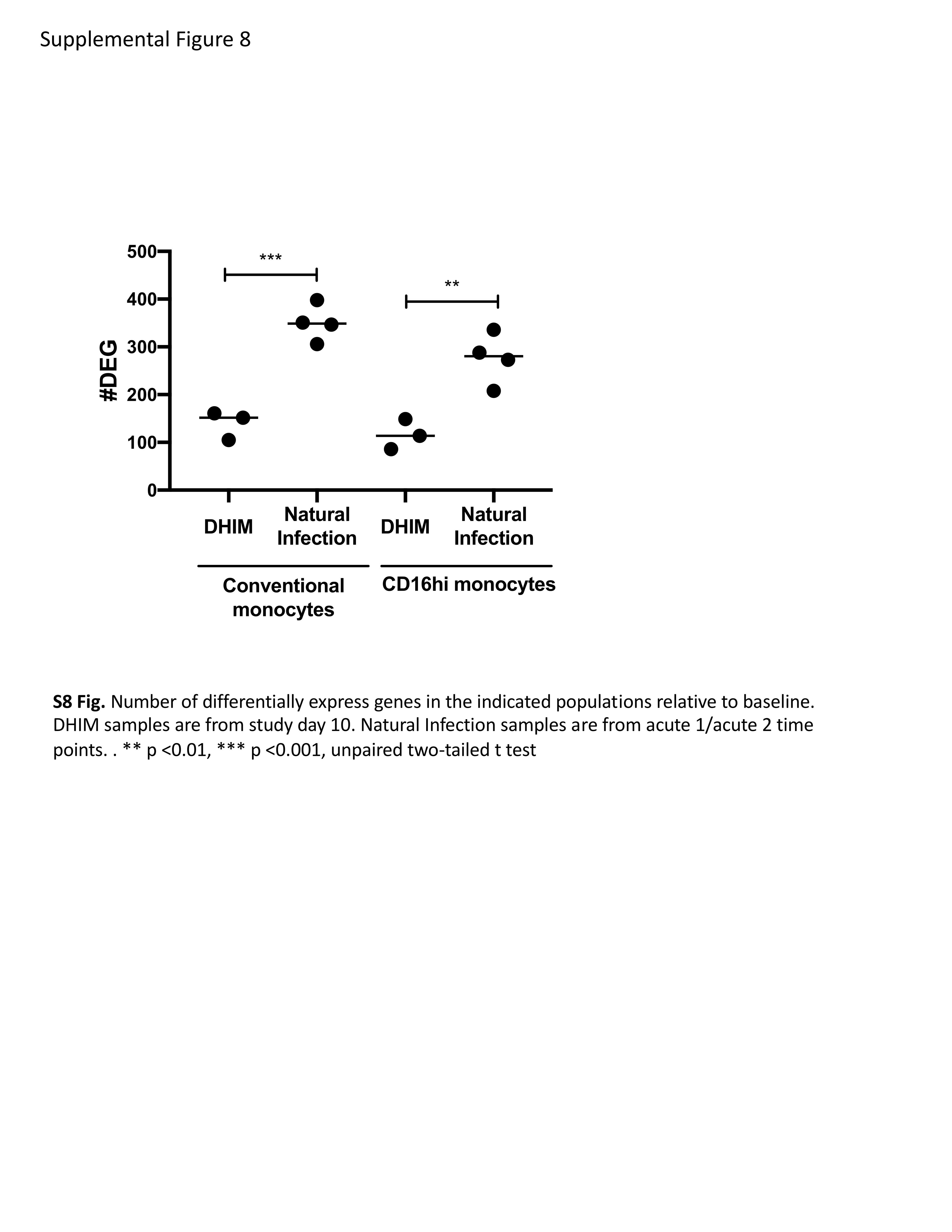

Supplement: S8 Fig — Experimental primary DENV-1 samples are from study day 10. Natural primary DENV-1 infection samples are from acute 1/acute 2 time points ** p <0.01, *** p <0.001, unpaired two-tailed t test. (TIF) [file ppat.1009240.s008.tif]
