# Supplementary material for: Temporally integrated single cell RNA sequencing analysis of PBMC from experimental and natural primary human DENV-1 infections
Source: PLoS Pathog. 2021 Jan 29;17(1):e1009240. doi: 10.1371/journal.ppat.1009240 (PMC7875406; doi:10.1371/journal.ppat.1009240)
Supplement: S1 Table — (DOCX) [file ppat.1009240.s009.docx]

**S1 Table.** Natural primary DENV-1 infection **s**ubject information

| **Subject** | **Age / Sex** | **Infection** | **Serotype** | **Severity** | **Fever day**  **(Prior to defervescence)** | **Illness day**  **(since fever onset)** |
| --- | --- | --- | --- | --- | --- | --- |
|  |  |  |  |  |  |  |
| **Primary #1** | 9/M | Primary | DENV1 | DF | -3 | 4 |
|  |  |  |  |  | -2 | 5 |
|  |  |  |  |  | 180 | -- |
|  |  |  |  |  |  |  |
| **Primary #2** | 5/M | Primary | DENV1 | DF | -5 | 2 |
|  |  |  |  |  | -4 | 3 |
|  |  |  |  |  | 180 | -- |
