# Supplementary material for: Temporally integrated single cell RNA sequencing analysis of PBMC from experimental and natural primary human DENV-1 infections
Source: PLoS Pathog. 2021 Jan 29;17(1):e1009240. doi: 10.1371/journal.ppat.1009240 (PMC7875406; doi:10.1371/journal.ppat.1009240)
Supplement: S2 Table — (DOCX) [file ppat.1009240.s010.docx]

**S2 Table.** Sample sequencing metrics

| **Subject** | **Study day** | **Total reads** | **Recovered cells** | **Mean reads/cell** | **UMI/cell** | **Unique features/cell** | **Total features** |
| --- | --- | --- | --- | --- | --- | --- | --- |
| DHIM #2 | 0 | 469,544,606 | 5,204 | 90,228 | 6,660 | 1,796 | 17,907 |
| DHIM #2 | 2 | 457,252,410 | 4,001 | 114,285 | 6,754 | 1,811 | 17,403 |
| DHIM #2 | 4 | 525,271,235 | 6,048 | 86,850 | 5,339 | 1,521 | 17,847 |
| DHIM #2 | 6 | 469,788,165 | 6,046 | 77,702 | 5,732 | 1,645 | 17,922 |
| DHIM #2 | 8 | 407,460,368 | 6,289 | 64,789 | 5,138 | 1,477 | 17,319 |
| DHIM #2 | 10 | 501,489,087 | 4,150 | 120,841 | 5,293 | 1,611 | 17,051 |
| DHIM #2 | 15 | 456,124,963 | 4,917 | 92,765 | 7,112 | 2,095 | 17,494 |
| DHIM #2 | 28 | 447,848,025 | 6,767 | 66,181 | 5,323 | 1,709 | 17,776 |
|  |  |  |  |  |  |  |  |
| DHIM #3 | 0 | 483,342,365 | 8,446 | 57,227 | 6,584 | 1,896 | 18,318 |
| DHIM #3 | 2 | 436,966,440 | 6,426 | 68,000 | 6,587 | 1,845 | 17,752 |
| DHIM #3 | 4 | 545,710,915 | 7,062 | 77,274 | 6,801 | 1,934 | 18,046 |
| DHIM #3 | 6 | 530,778,776 | 9,549 | 55,585 | 6,514 | 1,870 | 18,433 |
| DHIM #3 | 8 | 457,507,268 | 6,448 | 70,953 | 6,813 | 1,943 | 17,683 |
| DHIM #3 | 10 | 485,332,264 | 8,350 | 58,124 | 7,067 | 2,056 | 18,014 |
| DHIM #3 | 14 | 460,390,651 | 8,697 | 52,937 | 7,058 | 2,092 | 18,140 |
| DHIM #3 | 28 | 374,634,551 | 12,666 | 29,578 | 5,470 | 1,671 | 18,347 |
|  |  |  |  |  |  |  |  |
| DHIM #5 | 0 | 415,606,235 | 3,534 | 117,602 | 6,524 | 1,897 | 17,287 |
| DHIM #5 | 2 | 435,634,393 | 5,498 | 79,235 | 5,914 | 1,775 | 17,789 |
| DHIM #5 | 4 | 420,675,942 | 4,109 | 102,379 | 5,567 | 1,679 | 16,921 |
| DHIM #5 | 6 | 378,723,340 | 3,863 | 98,039 | 5,773 | 1,708 | 16,971 |
| DHIM #5 | 8 | 440,433,309 | 3,424 | 128,631 | 6,155 | 1,737 | 17,074 |
| DHIM #5 | 10 | 421,623,137 | 6,431 | 65,561 | 5,390 | 1,775 | 17,618 |
| DHIM #5 | 14 | 427,483,659 | 5,221 | 81,878 | 5,841 | 1,982 | 17,458 |
| DHIM #5 | 28 | 414,736,158 | 3,869 | 107,195 | 5,055 | 1,522 | 16,726 |
|  |  |  |  |  |  |  |  |
| Primary #1 | Acute 1 | 917,183,458 | 1,993 | 460,202 | 4,403 | 1,640 | 15,693 |
| Primary #1 | Acute 2 | 652,219,383 | 3,852 | 169,320 | 4,621 | 1,590 | 16,256 |
| Primary #1 | 180 | 704,437,542 | 5,292 | 133,114 | 5,113 | 1,523 | 17,201 |
|  |  |  |  |  |  |  |  |
| Primary #2 | Acute 1 | 695,123,220 | 3,613 | 192,395 | 3,336 | 1,279 | 15,573 |
| Primary #2 | Acute 2 | 682,392,037 | 3,196 | 213,514 | 1,793 | 876 | 14,896 |
| Primary #2 | 180 | 683,419,691 | 6,247 | 109,400 | 4,295 | 1,381 | 16,873 |
