# Supplementary material for: Temporally integrated single cell RNA sequencing analysis of PBMC from experimental and natural primary human DENV-1 infections
Source: PLoS Pathog. 2021 Jan 29;17(1):e1009240. doi: 10.1371/journal.ppat.1009240 (PMC7875406; doi:10.1371/journal.ppat.1009240)
Supplement: S3 Table — (DOCX) [file ppat.1009240.s011.docx]

**S3 Table.** Sample/population frequency: T cell populations

| **Subject** | **Study day** | Naïve CD4 | Memory CD4 | CD4 T | Naïve CD8 | CD8 CM | CD8 EM | Treg | MAIT | Vd2 gdT | NK/NKT | ILC | Activ T |
| --- | --- | --- | --- | --- | --- | --- | --- | --- | --- | --- | --- | --- | --- |
| DHIM #2 | 0 | 1021 | 933 | 227 | 624 | 451 | 153 | 73 | 108 | 123 | 169 | 43 | 3 |
| DHIM #2 | 2 | 798 | 769 | 168 | 596 | 347 | 77 | 50 | 91 | 82 | 119 | 24 | 5 |
| DHIM #2 | 4 | 1317 | 1064 | 255 | 820 | 469 | 121 | 82 | 121 | 117 | 184 | 42 | 7 |
| DHIM #2 | 6 | 1106 | 1179 | 226 | 898 | 570 | 124 | 100 | 133 | 123 | 183 | 41 | 10 |
| DHIM #2 | 8 | 1172 | 1277 | 224 | 768 | 608 | 160 | 104 | 125 | 140 | 193 | 50 | 11 |
| DHIM #2 | 10 | 852 | 817 | 147 | 398 | 336 | 109 | 69 | 81 | 84 | 118 | 23 | 10 |
| DHIM #2 | 15 | 913 | 873 | 225 | 809 | 255 | 240 | 76 | 140 | 94 | 467 | 43 | 196 |
| DHIM #2 | 28 | 1261 | 1080 | 219 | 669 | 467 | 209 | 100 | 162 | 131 | 326 | 49 | 28 |
|  |  |  |  |  |  |  |  |  |  |  |  |  |  |
| DHIM #3 | 0 | 963 | 1373 | 298 | 241 | 1068 | 1273 | 59 | 125 | 255 | 260 | 35 | 17 |
| DHIM #3 | 2 | 745 | 825 | 164 | 156 | 699 | 1290 | 50 | 132 | 286 | 351 | 41 | 19 |
| DHIM #3 | 4 | 815 | 937 | 196 | 166 | 798 | 1364 | 43 | 133 | 326 | 363 | 45 | 20 |
| DHIM #3 | 6 | 949 | 1114 | 227 | 213 | 1071 | 1857 | 52 | 165 | 412 | 485 | 57 | 24 |
| DHIM #3 | 8 | 626 | 725 | 166 | 149 | 788 | 1417 | 43 | 98 | 351 | 380 | 31 | 14 |
| DHIM #3 | 10 | 895 | 839 | 187 | 169 | 809 | 1687 | 46 | 121 | 392 | 439 | 36 | 101 |
| DHIM #3 | 14 | 860 | 688 | 162 | 149 | 892 | 1892 | 45 | 99 | 485 | 551 | 36 | 121 |
| DHIM #3 | 28 | 1216 | 1435 | 312 | 295 | 1529 | 2710 | 86 | 228 | 584 | 564 | 93 | 43 |
|  |  |  |  |  |  |  |  |  |  |  |  |  |  |
| DHIM #5 | 0 | 443 | 436 | 113 | 289 | 267 | 206 | 38 | 107 | 61 | 168 | 26 | 10 |
| DHIM #5 | 2 | 799 | 668 | 160 | 502 | 447 | 493 | 61 | 170 | 98 | 404 | 42 | 6 |
| DHIM #5 | 4 | 718 | 713 | 156 | 537 | 442 | 108 | 59 | 142 | 45 | 159 | 16 | 12 |
| DHIM #5 | 6 | 585 | 621 | 133 | 447 | 414 | 141 | 63 | 188 | 83 | 186 | 27 | 8 |
| DHIM #5 | 8 | 433 | 570 | 92 | 375 | 374 | 100 | 58 | 227 | 102 | 131 | 16 | 11 |
| DHIM #5 | 10 | 959 | 763 | 172 | 551 | 445 | 403 | 69 | 167 | 63 | 432 | 32 | 17 |
| DHIM #5 | 14 | 837 | 582 | 183 | 395 | 341 | 607 | 53 | 170 | 109 | 583 | 49 | 99 |
| DHIM #5 | 28 | 475 | 654 | 111 | 331 | 399 | 141 | 67 | 245 | 110 | 157 | 19 | 18 |
|  |  |  |  |  |  |  |  |  |  |  |  |  |  |
| Primary #1 | Acute 1 | 308 | 374 | 89 | 122 | 171 | 123 | 34 | 36 | 50 | 36 | 13 | 52 |
| Primary #1 | Acute 2 | 521 | 561 | 136 | 306 | 289 | 351 | 35 | 55 | 123 | 185 | 16 | 88 |
| Primary #1 | 180 | 747 | 588 | 159 | 301 | 355 | 308 | 71 | 94 | 81 | 191 | 31 | 63 |
|  |  |  |  |  |  |  |  |  |  |  |  |  |  |
| Primary #2 | Acute 1 | 83 | 140 | 32 | 51 | 104 | 254 | 22 | 41 | 44 | 162 | 15 | 29 |
| Primary #2 | Acute 2 | 258 | 342 | 65 | 99 | 434 | 591 | 23 | 64 | 79 | 287 | 18 | 24 |
| Primary #2 | 180 | 1111 | 583 | 252 | 471 | 495 | 855 | 45 | 67 | 120 | 344 | 35 | 30 |
