# Supplementary material for: Temporally integrated single cell RNA sequencing analysis of PBMC from experimental and natural primary human DENV-1 infections
Source: PLoS Pathog. 2021 Jan 29;17(1):e1009240. doi: 10.1371/journal.ppat.1009240 (PMC7875406; doi:10.1371/journal.ppat.1009240)
Supplement: S4 Table — (DOCX) [file ppat.1009240.s012.docx]

**S4 Table.** Sample/population frequency: B cells and myeloid linage cells

| **Subject** | **Study day** | Naïve B | Memory B | Plasmablast | Conventional mono | CD16hi mono | mDC | pDC | Mono platelet | Mega | Neutrophil | doublet |
| --- | --- | --- | --- | --- | --- | --- | --- | --- | --- | --- | --- | --- |
| DHIM #2 | 0 | 316 | 210 | 19 | 435 | 65 | 40 | 52 | 29 | 81 | 9 | 20 |
| DHIM #2 | 2 | 224 | 165 | 15 | 263 | 66 | 38 | 51 | 16 | 23 | 4 | 10 |
| DHIM #2 | 4 | 429 | 346 | 29 | 348 | 66 | 55 | 59 | 27 | 71 | 14 | 5 |
| DHIM #2 | 6 | 346 | 281 | 30 | 392 | 89 | 56 | 69 | 26 | 40 | 15 | 9 |
| DHIM #2 | 8 | 408 | 328 | 22 | 393 | 95 | 49 | 42 | 21 | 69 | 17 | 13 |
| DHIM #2 | 10 | 217 | 125 | 38 | 421 | 109 | 32 | 57 | 34 | 58 | 13 | 2 |
| DHIM #2 | 15 | 181 | 107 | 66 | 144 | 11 | 14 | 23 | 6 | 27 | 3 | 4 |
| DHIM #2 | 28 | 298 | 173 | 15 | 967 | 328 | 63 | 78 | 25 | 99 | 8 | 12 |
|  |  |  |  |  |  |  |  |  |  |  |  |  |
| DHIM #3 | 0 | 310 | 436 | 8 | 1067 | 227 | 97 | 35 | 146 | 106 | 14 | 33 |
| DHIM #3 | 2 | 192 | 213 | 9 | 818 | 192 | 47 | 46 | 79 | 42 | 19 | 11 |
| DHIM #3 | 4 | 238 | 302 | 5 | 876 | 212 | 50 | 31 | 65 | 47 | 14 | 16 |
| DHIM #3 | 6 | 330 | 395 | 7 | 1458 | 342 | 80 | 45 | 91 | 116 | 27 | 32 |
| DHIM #3 | 8 | 187 | 217 | 8 | 781 | 213 | 51 | 33 | 76 | 67 | 12 | 15 |
| DHIM #3 | 10 | 227 | 245 | 23 | 1324 | 614 | 59 | 39 | 39 | 38 | 10 | 11 |
| DHIM #3 | 14 | 178 | 143 | 29 | 1618 | 447 | 72 | 21 | 91 | 86 | 16 | 16 |
| DHIM #3 | 28 | 481 | 669 | 25 | 1512 | 385 | 82 | 61 | 108 | 169 | 21 | 58 |
|  |  |  |  |  |  |  |  |  |  |  |  |  |
| DHIM #5 | 0 | 202 | 111 | 6 | 712 | 175 | 51 | 27 | 46 | 27 | 3 | 10 |
| DHIM #5 | 2 | 325 | 134 | 6 | 760 | 234 | 56 | 33 | 51 | 22 | 14 | 13 |
| DHIM #5 | 4 | 355 | 125 | 7 | 311 | 67 | 70 | 7 | 36 | 15 | 6 | 3 |
| DHIM #5 | 6 | 344 | 148 | 16 | 283 | 64 | 42 | 9 | 41 | 13 | 3 | 4 |
| DHIM #5 | 8 | 329 | 137 | 23 | 263 | 63 | 70 | 14 | 16 | 18 | 0 | 2 |
| DHIM #5 | 10 | 405 | 143 | 16 | 1225 | 327 | 65 | 51 | 63 | 26 | 10 | 27 |
| DHIM #5 | 14 | 253 | 63 | 32 | 595 | 128 | 28 | 38 | 25 | 43 | 4 | 4 |
| DHIM #5 | 28 | 500 | 167 | 12 | 238 | 63 | 56 | 6 | 15 | 73 | 4 | 8 |
|  |  |  |  |  |  |  |  |  |  |  |  |  |
| Primary #1 | Acute 1 | 236 | 133 | 92 | 73 | 20 | 6 | 10 | 0 | 12 | 0 | 3 |
| Primary #1 | Acute 2 | 328 | 354 | 164 | 141 | 34 | 10 | 115 | 5 | 29 | 2 | 4 |
| Primary #1 | 180 | 503 | 561 | 116 | 781 | 115 | 93 | 67 | 23 | 21 | 7 | 16 |
|  |  |  |  |  |  |  |  |  |  |  |  |  |
| Primary #2 | Acute 1 | 154 | 165 | 100 | 1664 | 389 | 35 | 80 | 33 | 13 | 1 | 2 |
| Primary #2 | Acute 2 | 153 | 109 | 51 | 331 | 159 | 7 | 51 | 14 | 35 | 0 | 2 |
| Primary #2 | 180 | 663 | 611 | 29 | 260 | 121 | 41 | 41 | 38 | 24 | 2 | 9 |
