# Supplementary material for: Temporally integrated single cell RNA sequencing analysis of PBMC from experimental and natural primary human DENV-1 infections
Source: PLoS Pathog. 2021 Jan 29;17(1):e1009240. doi: 10.1371/journal.ppat.1009240 (PMC7875406; doi:10.1371/journal.ppat.1009240)
Supplement: S5 Table — (DOCX) [file ppat.1009240.s013.docx]

**S5 Table.** Conserved differentially expressed genes: day 10 Experimental primary DENV-1 infection

| **Population** | **Induced core genes** | **Suppressed core genes** |
| --- | --- | --- |
| **Conventional monocytes** | IFITM1, IFI6, LY6E, IFITM3, ISG15, IFI44L, IFITM2, TYMP, XAF1, PSME2, RNF213, SIGLEC1, NCF1, TNFSF10, LAP3, APOBEC3A, MT2A, PLAC8, SERPING1, UBE2L6, TNFSF13B, IRF7, PSMB9, IFI35, PARP14, VAMP5, MX1, EPSTI1, TRIM22, EIF2AK2, OAS1, IFI44, RSAD2, GBP1, OAS3, HBB, WARS, STAT1, TMEM123, MX2, STAT2, PLSCR1, ISG20, SAMD4A, HES4, OASL, TXNIP | IL1B, CXCL8, RPS3A, RPL4, EEF2, RPL6, RPL5, EIF3L, RPL3, EIF4B |
| **CD16^hi^ monocytes** | IFITM1, IFITM3, LY6E, IFI6, APOBEC3A, IFI44L, RSAD2, TNFSF10, TYMP, ISG15, BST2, UBE2L6, XAF1, LAP3, TNFSF13B, NCF1, PLAC8, IFI35, PSMB9, EPSTI1, IFIT2, TRIM22, PLSCR1, VAMP5, PSME2, SERPING1, IRF7, MX1, PARP14, EIF2AK2, WARS, IFI44, MX2, CXCL10, HES4, GBP1, TMEM123, OAS3, HERC5, OAS1 | MT-CYB, IL1B, RPL3, MT-ND4, RPL10A, AHNAK |
| **mDC** | IFI6, IFITM1, LY6E, IFITM3, IFI44L, IFITM2, ISG15, UBE2L6, PSME2, TYMP, PLAC8, XAF1, MX1, STAT1, PSMB9, EPSTI1, LAP3, MT2A, IRF7, LGALS9, NAPA, MX2 | EEF1B2, EIF3L, RPL5 |
| **pDC** | LY6E, PLAC8, ISG15, BST2, IFI44L, IFITM2, COX5A, IFI35 | -- |
| **MAIT** | IFITM1, IFI6, LY6E, IFITM3, IFITM2, IFI44L, BST2, ISG20, ISG15, XAF1, PSME2, TRIM22, PSMB9, STAT1, IRF7, TYMP, IFI35, EPSTI1 | -- |
| **ILC** | IFITM1, IFI6, IFI44L, LY6E | -- |
| **NK/NKT** | IFI6, IFITM1, LY6E, IFI44L, XAF1, IFITM3, BST2, IFITM2, ISG15, PSME2, PLSCR1, IFI35, TRIM22, SHISA5, ISG20, MX2, EIF2AK2, UBE2L6, PSMB9, EPSTI1, MX1, IRF7, PARP9, TYMP, STAT1, LGALS1 | KLRB1 |
| **Vδ2 γδ T** | IFITM1, IFI6, LY6E, XAF1, ISG20, IFI44L, IFITM2, ISG15, STAT1, TRIM22, BST2, PLSCR1, EPSTI1, IFI35, MX2, EIF2AK2, PSME1, IRF7 | KLRB1 |
| **Naïve B** | IFITM1, IFI6, IFI44L, XAF1, LY6E, ISG15, EIF2AK2, IRF7, IFITM2, ISG20, TRIM22, BST2, MX2, RNF213, MX1, STAT1, PLSCR1, DRAP1, PSMB9, EPSTI1, PSME2 | -- |
| **Memory B** | IFI6, IFITM1, LY6E, ISG15, IFITM3, ISG20, XAF1, BST2, MX1, IFITM2, TRIM22, IFI44L, EIF2AK2, PSME2, STAT1, IRF7, MX2 | -- |
| **Naïve CD4** | IFITM1, IFI6, IFI44L, LY6E, HBB, STAT1, XAF1, TRIM22, ISG15, EIF2AK2, SP100, EPSTI1, PLSCR1, BST2, ISG20 | -- |
| **Memory CD4** | **I**FI6, IFITM1, LY6E, IFI44L, ISG15, XAF1, BST2, EIF2AK2, ISG20, TRIM22, STAT1, IFITM2, PSMB9, IFI44, EPSTI1, SP100, PARP9, PLSCR1, MX2 | -- |
| **Naïve CD8** | IFI6, IFITM1, IFI44L, LY6E, STAT1, TRIM22, BST2, ISG15, ISG20, XAF1, PSME2, IFI44, EIF2AK2, IFITM2 | -- |
| **CD8 CM** | IFI6, IFITM1, LY6E, IFI44L, XAF1, BST2, TRIM22, EIF2AK2, STAT1, ISG20, ISG15, SP100, PSMB9, EPSTI1, PLSCR1 | -- |
| **CD8 EM** | IFI6, IFITM1, LY6E, BST2, ISG15, IFI44L, PSME2, ISG20, STAT1, IFITM3, TRIM22, IFITM2, PLSCR1, IRF7, MX1, PSMB9, EIF2AK2, XAF1, IFI35, MT2A, SP100, DRAP1, MX2 | -- |
| **Treg** | IFI6, IFITM1, LY6E, ISG15, SP100, TRIM22, EIF2AK2, DRAP1 | RPL4 |
