# Supplementary material for: Temporally integrated single cell RNA sequencing analysis of PBMC from experimental and natural primary human DENV-1 infections
Source: PLoS Pathog. 2021 Jan 29;17(1):e1009240. doi: 10.1371/journal.ppat.1009240 (PMC7875406; doi:10.1371/journal.ppat.1009240)
Supplement: S6 Table — (DOCX) [file ppat.1009240.s014.docx]

**S6 Table.** Core differentially expressed genes: natural primary DENV-1 infection

| **Population** | **Induced core genes** | **Suppressed core genes** |
| --- | --- | --- |
| **Conventional monocytes** | IFIT3, IFI27, IFIT1, RSAD2, USP18, IFI44L, OASL, SERPING1, CCL2, OAS3, IFIT2, IFITM1, MX1, HERC5, ISG15, ZBP1, GBP1, SIGLEC1, XAF1, CMPK2, IFI6, LGALS3BP, SAMD9L, ISG20, PARP9, OAS2, EPSTI1, IFI44, IFIH1, STAT1, LY6E, SPATS2L, CXCL10, IRF7, OAS1, HLA-A, PARP12, EIF2AK2, PARP14, DDX58, TNFSF10, MX2, DDX60L, RNF213, GBP5, GBP4, TRIM22, SAMD9, PLAC8, CTSL, IFI35, MT2A, IFI16, NT5C3A, HLA-B, APOBEC3A, HLA-C, TCN2, IFITM2, HSH2D, LAP3, SP110, GIMAP4, IFITM3, SAMD4A, XRN1, SELL, RNASE2, NMI, FCGR1A, BST2, SMCHD1, PML, PSME2, PSMB9, PLSCR1, UBE2L6, GCH1, VAMP5, PHF11, TMEM123, TYMP, DRAP1, NAPA, SCO2, CCR1, LGALS9, IL1RN, PSMA4, MYL12A, MARCKS, MAFB | TPT1, RPS28, EEF1A1, RPS13, RPS3A, RPLP1, RPL8, RPL18, RPS7, RPS14, RPL34, RPS24, RPL35A, RPS15A, MT-ND3, MT-CO3, RPS8, RPS4X, RPL11, RPL10, RPL19, RPL5, RPL6, RPL9, NACA, RPL22, RPL26, RPL37, RPS23, RPL15, RPL7A, RPL32, RPL18A, BTF3, RPL3, RPL30, RPLP2, RPS16, RPS27A, RPS6, RPLP0, RPL29, COX4I1, PABPC1, CSTA, COTL1, SLC25A6, RPL4, RPL27, RPL13, RPL10A, RPS5, EEF1B2, RPL21, AP1S2, RPS3, EEF2, GSTP1, RPS18, PCBP2, RPL7, EIF3L, GNB2L1, SLC25A5, RPS4Y1, ALDH2, EIF3F, RBM3, FCGRT, EIF3E, EIF3H, CRTAP |
| **CD16^hi^ monocytes** | IFI44L, IFIT1, OASL, RSAD2, SIGLEC1, IFI27, IFIT3, MX1, IFI44, IFIT2, CXCL10, XAF1, HERC5, LGALS3BP, ISG15, SERPING1, DDX60L, OAS3, PLSCR1, USP18, MX2, APOBEC3A, EIF2AK2, TNFSF10, IRF7, FFAR2, CD300E, ZBP1, EPSTI1, GBP4, NCF1, TCN2, GLUL, IFI35, DDX58, SAMD9L, LGALS9, HLA-A, IFIH1, SPATS2L, TRIM22, IFI6, MARCKS, CDKN1A | RPL6, RPS3A, EEF1A1, RPS15A, RPL10, RPS13, RPS8, RPS23, RPL34, RPS4X, RPS27A, RPL8, RPL14, RPS14, RPS7, RPL18A, RPS6, RPL12, RPLP2, RPL35A, RPL18, RPL11, RPL21, RPL7A, RPL9, RPL5, SLC25A6, RPL32, RPL10A, RPS24, RPS21, RPL13, RPL30, RPL4, MT-CO3, RPL19, RPL22, RPS16, RPL26, RPL15, GNB2L1, COTL1, PABPC1, RPL3, SLC25A5, ACTG1, EEF2, EEF1B2, NACA, RPLP0, RPL27, RPS25, RPS5, TPT1, RPL23A, RPS3, RPSA, RPS18, PFDN5, EIF3K, RPL37A, UQCRB, BTF3, RPL29, MT-ND3, MT-CYB, RPS4Y1, RPL23, YBX1, CCNI, RPS17, MT-ND4, NAP1L1, EIF3L, RBM3 |
| **mDC** | IFI27, USP18, IFIT3, RSAD2, HERC5, IFI44L | -- |
| **pDC** | IFI6, IFI44L, IFITM1, ISG15, ISG20, MX1, IFITM2 | -- |
| **MAIT** | IFI44L, IFI6, MX1, ISG15, MX2, IRF7, LY6E, IFITM1, IFI44, OAS1, IFIT1, IFIT3, MT2A, RSAD2, IFI35, OASL, PLSCR1, XAF1, ISG20, OAS3, BST2, TRIM22, UBE2L6, DRAP1, IFITM3 | RPS27A, EEF1A1, RPS4X, RPL3, RPS8, RPL13, MT-CYB, RPS3A, RPS14, RPS3, RPS6, MT-CO3, RPLP2, RPL10, MT-ND3, RPL21, MT-ATP6, RPL5, MT-ND5, RPL4 |
| **ILC** | IFI44L, IFI6, IFITM1 | -- |
| **NK/NKT** | IFI44L, IFIT3, MX1, IFI6, OAS1, OAS3, IFIT1, RSAD2, PLSCR1, IRF7, ISG15, OASL, MX2, EPSTI1, PARP9, ISG20, LY6E, IFI35, XAF1, IFI44, DTX3L, LGALS9, OAS2, MT2A, STAT1, TYMP, BST2, SAMD9L, EIF2AK2, ADAR, RNF213, SP100, SAMD9, GBP1, TRIM22, IFITM1, NT5C3A, CD38, PARP12, LAG3, UBE2L6, PRF1, ZBP1, S100A11, WARS, IFITM3, IFI16 | EEF1A1, RPS14, RPL13, RPL3, RPS3A, MT-CO3, RPS27A, MT-ATP6, RPS4X, RPL21, RPS8, MT-CYB, RPL5, MT-ATP8, KLRB1 |
| **Vδ2 γδ T** | IFI44L, IFI6, ISG15, LY6E, MX1, IFITM1, MT2A, IRF7, XAF1, IFIT3, RSAD2, IFITM3, OAS1, ISG20, CMPK2, STAT1, MX2, RNF213, UBE2L6, BST2, LAG3, SAMD9L, PLSCR1, SAMD9, IFITM2, TYMP, PSME2 | RPS27A, MT-CYB, RPS14, RPS3A, RPL5, RPL3, RPL21, EEF1A1, RPS3, RPS4X, RPLP2, RPS8, MT-CO3, EEF1B2, MT-ND4L, MT-ND5 |
| **Naïve B** | IFI44L, IFITM1, XAF1, ISG15, IFI6, IFIT3, MX1, IFIT1, MX2, IRF7, LY6E, PLSCR1, ISG20, HLA-A, CMPK2, EPSTI1, SAMD9L, OAS1, STAT1, UBE2L6, EIF2AK2, TRIM22, IFITM2, IFITM3, DRAP1, IFI44, HERC5, BST2, SP110, SAMD9, RNF213, IFI35, ZBP1, PARP9, PSME2, PSMB9, IFI16, MT2A, PLAC8, COX5A, STAT2, CHMP5 | RPS8, RPS3A, RPL3, RPS6, MT-CO3, RPL10A, RPL21, MT-CYB, MT-ATP6, RPL4, EIF3L |
| **Memory B** | IFIT3, IFI6, IFIT1, XAF1, ISG15, MX1, IRF7, IFI44L, MX2, IFITM1, HERC5, LY6E, STAT1, OAS1, PLSCR1, EIF2AK2, SAMD9L, ISG20, PARP9, EPSTI1, BST2, TRIM22, CTA-384D8.34, RSAD2, UBE2L6, MT2A, OAS2, RNF213, IFITM2, IFI35, ZBP1, SAMD9, IFITM3, DRAP1, PPM1K, DDX60L, TYMP, SAT1, CLEC2D, FCRL5 | RPS8, RPS3A, RPS6, RPS4X, RPL3, RPL21, RPL10A, RPL27, MT-CO3, MT-CYB, RPL4, EIF3L |
| **Naïve CD4** | IFI44L, ISG15, MX1, XAF1, IFIT3, LY6E, IRF7, HLA-A, IFI6, IFI44, OAS1, IFITM1, MX2, STAT1, EIF2AK2, PLSCR1, MT2A, EPSTI1, IFI35, BST2, TRIM22, RNF213, UBE2L6, DRAP1, IFI16, SMCHD1, MYL12A, C19orf66, ISG20, PSME2, PARP10, SAT1, IRF1 | RPS14, RPS3A, RPS6, RPL3, RPS27A, RPL21, RPL4, RPS16, MT-CYB, EIF3L |
| **Memory CD4** | IFI44L, ISG15, MX1, IFI6, IFIT3, XAF1, LY6E, IRF7, MX2, HLA-A, OAS1, IFITM1, MT2A, HLA-B, EPSTI1, HLA-E, STAT1, RSAD2, EIF2AK2, IFI44, ISG20, OASL, PLSCR1, SAMD9L, TRIM22, BST2, SAMD9, SP100, PARP9, RNF213, OAS3, HERC5, GBP1, IFI35, TYMP, SP110, UBE2L6, TNFSF10, IFI16, PHF11, IER2, DRAP1, ADAR, PSMB9, LGALS9, ZBP1, PARP12, PSME2, SMCHD1, PARP10, IFITM3 | EEF1A1, RPS3A, RPS27A, RPL13, RPS8, RPL34, RPS14, RPL3, RPS4X, RPS6, RPS18, RPL10, TPT1, RPL18, RPL21, RPL5, RPLP2, RPS5, RPL6, RPL10A, RPL4, RPL27, RPS16, MT-CYB, MT-CO3, EEF2, EIF3L |
| **Naïve CD8** | IFI44L, ISG15, MX1, XAF1, IFI6, IRF7, LY6E, IFIT3, IFI44, STAT1, IFITM1, EPSTI1, EIF2AK2, MT2A, OAS1, MX2, PLSCR1, BST2, SAMD9, IFI35, TRIM22, MYL12A, RSAD2, PARP10, RNF213, DRAP1, IFI16, SAMD9L, IFITM3, ISG20, PSMB9 | RPS6, RPS27A, RPS3A, RPL3, RPL21, MT-CO3, RPL4, RPS16, MT-CYB, MT-ATP6, EIF3L |
| **CD8 CM** | IFI44L, ISG15, MX1, IFI6, XAF1, LY6E, OAS1, IFITM1, IRF7, IFIT3, EIF2AK2, MX2, PTPRCAP, RSAD2, ISG20, EPSTI1, BST2, IFI35, STAT1, SP100, PLSCR1, MT2A, PARP9, OASL, RNF213, OAS2, TYMP, TRIM22, SAMD9, SAMD9L, DRAP1, IFI16, UBE2L6, IFITM3, PSME2, ZBP1, PSMB9, PARP10, LAG3 | RPS4X, MT-CYB, RPS3A, RPS27A, RPL3, EEF1A1, RPL13, RPS14, MT-CO3, RPL21, RPS6, RPS8, RPLP2, RPS16, RPL5, RPL4, MT-ATP6, MT-ND4L, MT-ND5, EIF3L, EEF2 |
| **CD8 EM** | MX1, IFI6, ISG15, IFI44L, XAF1, IFIT3, IFIT1, RSAD2, EIF2AK2, OAS1, MX2, ISG20, LY6E, IRF7, STAT1, MT2A, IFITM1, IFI35, PARP9, EPSTI1, BST2, IFI44, GBP1, PLSCR1, LAG3, UBE2L6, RNF213, TYMP, OASL, LGALS9, OAS2, SP100, PSME2, SAMD9L, TRIM22, HERC5, SP110, ADAR, TAP1, SAMD9, PSMB9, PRF1, IFI16, CD38, DRAP1, S100A11, ZBP1, IFITM3, PARP10 | RPS27A, RPS3, RPS14, RPL13, MT-CYB, RPS3A, RPL3, RPS4X, RPL7A, MT-CO3, EEF1A1, RPL21, RPL5, RPS8, RPS6, MT-ATP6, MT-ND4L, RPL23A, MT-ND4, MT-ND5, MT-CO2, MT-ATP8, RPL4, HOPX, EIF3L |
| **Treg** | MX1, IFI6, IFIT3, ISG15, LY6E, BST2, IFITM1, IFITM3 | RPS3A, EEF1A1, RPS4X, RPL5, RPL3, EEF2, RPL4 |
