# Supplementary material for: Temporally integrated single cell RNA sequencing analysis of PBMC from experimental and natural primary human DENV-1 infections
Source: PLoS Pathog. 2021 Jan 29;17(1):e1009240. doi: 10.1371/journal.ppat.1009240 (PMC7875406; doi:10.1371/journal.ppat.1009240)
Supplement: S7 Table — (DOCX) [file ppat.1009240.s015.docx]

**S7 Table.** Antibodies used for flow cytometry

| **Antibody** | **Manufacture** | **Clone** | **Cat#** | **Lot#** | **Dilution used** |
| --- | --- | --- | --- | --- | --- |
| CD8 PerCP Cy5.5 | BD | SK1 | 341051 | 4099943 | 1:20 |
| Ki67 AF488 | BD | B56 | 561165 | 6261744 | 1:20 |
| CD38 PE | Biolegend | HB7 | 356604 | B178346 | 1:160 |
| CD38 BV421 | Biolegend | HIT2 | 303526 | B211387 | 1:20 |
| CD4 BV605 | Biolegend | RPA-T4 | 300556 | B232691 | 1:160 |
| CD19 BV785 | Biolegend | HIB19 | 302240 | B211586 | 1:160 |
| CD3 BV785 | Biolegend | OKT3 | 317330 | B231963 | 1:160 |
| IgG PE-CF594 | BD | G18-145 | 562538 | 6119766 | 1:40 |
| CD303 PeCy7 | Biolegend | 201A | 354214 | B239872 | 1:160 |
| IgA APC | Miltenyi Biotec | REA1014 | 130-116-879 | 5181030090 | 1:160 |
| CD14 AF700 | BD | M5E2 | 557923 | 7047605 | 1:80 |
| CD3 APC-Cy7 | BD | SP34-2 | 557757 | 7025869 | 1:160 |
| CD56 APC Cy-7 | Biolegend | HCD56 | 318332 | B178913 | 1:160 |
| CD56 PeCy7 | BD | B159 | 557757 | 5163892 | 1:20 |
| HLA-DR FITC | BD | G46-6 | 555811 | 28731 | 1:40 |
| CD16 PerCP-ef710 | eBioscience | CB16 | 46-0168-42 | 1991932 | 1:320 |
| IgD BV510 | Biolegend | 1A6-2 | 348220 | B226970 | 1:40 |
| IgM BV605 | BD | G20-127 | 562997 | 7026708 | 1:40 |
| CD1c BV650 | BD | F10/21A3 | 742749 | 8305935 | 1:160 |
| CD11c BUV395 | BD | B-ly6 | 563787 | 8141967 | 1:160 |
| CD27 BUV737 | BD | L128 | 564301 | 8164573 | 1:40 |
