# Supplementary material for: Temporally integrated single cell RNA sequencing analysis of PBMC from experimental and natural primary human DENV-1 infections
Source: PLoS Pathog. 2021 Jan 29;17(1):e1009240. doi: 10.1371/journal.ppat.1009240 (PMC7875406; doi:10.1371/journal.ppat.1009240)
Supplement: Attachment — Submitted filename: Response_to_reviewers_PloSPath.pdf [file ppat.1009240.s016.pdf]

Part I - Summary

<u>**Reviewer #1**</u>:

Technically, the authors downsampled the RNA data (which is important) but it is not explicitly stated that downsampling was to the same number of reads per cell in the analysis. This is critical.

Downsampling scRNAseq data for the purpose of data normalization and stabilization is the approach utilized by the 10x Genomics Cellranger analysis software package. However, for this study we utilized the R package Seurat for multi-sample integration and data normalization, specifically the "scTransform" and data-integration functions of the software package.

The "scTransform" function leverages a regularized negative binomial regression model where cellular sequencing depth is utilized as a covariate, thereby removing the influence of technical variation (such as sequencing depth) from downstream analyses while maintaining biological heterogeneity across the samples [1]. This alleviates the need to downsample datasets to correct for differences in sequencing depth. The sequencing depth (reads/cell) for each sample is shown in Supplemental Table 2.

We have added additional language to the text of the manuscript and to the Methods section emphasizing these analysis details, as well as a specific reference for the scTransform data normalization tool used in the Seurat package.

Figure 1 provides information on the three subjects from the DHIM group but the information on the two children who had natural infection is in the supplementary data (supplementary Table 1 and supplementary figure 1). These data all need to be in figure 1.

We have moved the indicated figure from Supplementary Figure 1 to Figure 1B of the revised manuscript.

The authors need to explain why the IgG/IgM values are different between the two groups.

As noted in the Methods, these subjects (DHIM versus natural infection) were studied under two distinct clinical study protocols conducted in different geographical locations. The assays used to assess DENV-specific serum IgG/IgM levels in the two study arms were different. For this reason, we use this information only to provide a general reference point for the timing of immune responses to viral infection. We have added additional text to the Results and Methods sections emphasizing and describing these technical differences.

It would be very helpful to show viremia data for the three timepoints of the two natural infection subjects. I say this as it is clear the best data for the DHIM group comes from the peak viremia samples (day 10), and viremias are very similar titers and this point is made on line 284 by the authors. However, the parameters of the samples from the two natural infections need to be stated, in particular viremia data on the timepoints examined.

As noted above, the subjects with natural DENV infection were recruited under a different study protocol than the DHIM subjects. Plasma DENV RNA levels were not measured in the natural infection cases, and plasma samples were not available to SUNY for this analysis. We understand the reviewer's interest in these data; however, there are several reasons why this information would not be helpful to interpret our findings. First, previous data from studies of natural DENV infection conducted by us and others have shown that viremia titers typically are at or near the highest level at the onset of symptoms

and decline rapidly thereafter. Based on the timing of sample collection relative to the onset of symptoms in our subjects (data added to the revised Supplemental Table 1), we believe that the natural infection samples studied do not correspond to the peak of viremia, and that viremia data would not be able to exclude a significant difference in viremia kinetics from the challenge strain. Second, as noted in the manuscript, DHIM subjects were challenged with a partially attenuated strain of DENV administered via needle inoculation. In the unlikely scenario that viremia kinetics of DHIM and natural infection were entirely equivalent, we would be unable to exclude other significant differences, such as intracellular replication kinetics or tissue distribution. We used the viremia data in DHIM subjects along with IgM and IgG data principally to draw parallels between sample collection timepoints in the DHIM and natural infection cases. We believe that our conclusions take these issues into account.

The authors do not discuss why these samples are the best in the Discussion. This is needed as lines 361-362 in the Discussion are very vague. The possibilities that the viremias for the natural infections are not equivalent to those in the DHIM samples needs to be considered.

As noted above, we agree with the reviewer that the viremias of the natural infection and DHIM arms of the study may not be equivalent and that this difference could contribute to the observed differential gene expression profile. We also acknowledge that time points that were not tested might yield somewhat different results and conclusions. The objective of this study was to quantify the differential immunological/transcriptional profiles associated with either natural DENV infection or DHIM, irrespective of the source of variation (viral genetics, viremia level, etc).

Why use only three DHIM subjects and two with natural infections. How was selection of subjects made? Does the difference in ages of subjects in the two groups contribute to the differences in results?

The DHIM subjects were selected for this analysis as they exhibited similar viremia and seroconversion kinetics and were representative of the SUNY/WRAIR DHIM-1 study as a whole. While we sincerely wish that additional subjects could have been included in this analysis, the cost and complexity of the scRNAseq assay required us to make some pragmatic decisions.

The differences in the age of the subjects included in either the DHIM or natural infection arms of the study is certainly a significant confounder in our analysis. However, primary dengue infections are almost exclusively observed in children in dengue endemic areas (such as Thailand), so age matching the two arms of this study was not possible.

We have added additional language to the manuscript explaining the selection criteria used for the DHIM subjects, and the necessity for the age difference in the two study arms.

The paper needs a summary figure to provide an overview for readers for a very complex paper. Currently, there is a supplementary figure 9. This needs to be moved to the main paper and expanded.

We have moved Supplementary Figure 9 to Figure 5E and have expanded the information contained therein.

Presumably there are viral genome differences between the attenuated DENV-1 in the DHIM and the viruses in the natural infection. If possible, the sequence differences (NT and deduced AA) between these viruses needs to be included in the text to help understand the viral determinants for the differences between the two types of infection.

The reviewer raises an interesting point. Unfortunately, we do not have the DENV-1 sequences from the natural infections. Furthermore, the wild-type DENV-1 parent of the DHIM1 vaccine strain undoubtedly contributed a substantial number of nucleotide and amino acid sequence differences

compared to the wild-type DENV-1 viruses from the natural infections. Therefore, even if we had the DENV-1 sequences from the natural infections, it would be difficult to disentangle the contribution of natural sequence variation and attenuating mutations.

Furthermore, it is disappointing that the authors have not speculated on the molecular basis of attenuation of the DHIM virus. This is a potentially important spinoff of the paper.

The nucleotide and deduced amino acid sequence differences between the DHIM1 vaccine strain and its wild-type parent, DENV-1 Nauru/West Pac/1974 isolate, were determined previously [2]. However, the mechanism of attenuation of the vaccine strain was never elucidated, which is in large part due to inadequate animal models for dengue. For human infections, the most informative comparison would have been between the DHIM-1 vaccine strain and its wild-type parent and not natural infections with other DENV-1 strains, as was done here. It is for this reason and also what is stated in the response above that it would be very difficult to describe the molecular basis of attenuation of the DHIM1 strain.

Lines 329-330 of the Discussion are vague. Clearly, there is "insights" from the results but these need to be spelt out.

We have added additional text to this section of the Discussion better explaining our thoughts on this aspect of our study.

<u>**Reviewer #2**</u>:

This study provides information on the transcriptional response of peripheral blood cells during dengue infection at a single cell level and would be a valuable tool for mining the transcriptional profile for new hypotheses relating to dengue infection control and severity. However, there are some drawbacks to the study design.

While I am sympathetic to the cost of the study and the uniqueness of the information provided by this sequencing technique, a significant drawback is the limited numbers of patients from which the data are derived, the mis-matched age comparisons and, as a consequence, the limited robustness of the statistical plan.

We agree with the reviewer that the limited number of subjects included in both arms of the study is a significant drawback. During the design phase of this project we had to weigh including more subjects in the analysis against analyzing more timepoints per subject. We made the decision to focus on the temporal aspect of DENV-elicited inflammation, as we felt that detecting the earliest phase of DENV-elicited inflammation was a key goal of the study. We have tried to stress these limitations in the discussion section of the manuscript.

Although limited in N of total patients, the numbers of time points of longitudinal analysis are an advantage of the study. Overall, the conclusions of the study might be more strong if the focus of the study shifted to the longitudinal changes in paired samples through the course of experimental infection, as emphasized by the title and nicely presented in Fig. 3. This could also be more clearly defined using the N available rather than comparisons between 2 and 3 patient samples per group for the natural versus controlled infection description where differences in IFN and protein translation pathways are reported. In short, this is a valuable data set but the authors should be more cautious which conclusions can be drawn from it and not all conclusions are well supported. There is also an incomplete description of the corresponding infection kinetics (for the natural infection group) which would allow better interpretation of the data.

We thank the reviewer for their comments

__Reviewer #3__: *Here, 3 experimental human infections with live-attenuated DENV-1 are compared with 2 cases of natural DENV-1 infections by scRNAseq. Very similar inflammatory differential gene regulation was observed. Natural infection was associated with a more pronounced suppression of translation and mitochondrial genes in monocytes, consistent with increased replication of the virus.*

We thank the reviewer for their succinct and accurate summary of our study.

Part II – Major Issues: Key Experiments Required for Acceptance

__Reviewer #1__:

No new experiments are needed but a lot of clarifications are needed to interpret the paper.

We thank the reviewer and have endeavored to clarify the points indicated above.

__Reviewer #2:__

The terminology of "controlled" DENV infection see a little non-standard and I would suggest reserving for the discussion. I would suggest considering other alternatives to convey that this is not a fully virulent challenge model. This experimental DENV infection is with an attenuated vaccine candidate, right? Might this be the effect described be of virus attenuation only rather than the same gene patterns that regulate natural infection control of non-attenuated strains? I think this caveat needs to be discussed further and the abstract/intro should be more cautiously worded regarding what the experimental set up can establish.

We agree with the reviewer that the attenuating mutations present in the virus strain used in the challenge model arm of the study contribute significantly to the differential transcriptional response observed relative to a natural DENV infection. Indeed, the intent of this study was to examine and quantify the presumably distinct immune transcriptional profile associated with either natural or experimental/attenuated DENV infection. We have added additional text to the manuscript emphasizing that the divergent transcriptional responses observed between the two arms of the study are likely attributable to the attenuation mutations present in the challenge virus, as well as differences attributable to different methods of virus delivery.

One important distinction in the comparison is that samples from adults and children are compared. It would have been much stronger if the Thai natural infection samples had been case controlled and aged matched. Also the N for the natural infection is only 2 which is not really sufficient for making broad comparisons. It would have been much better to include a few additional samples. Is this possible? If not, the study is still informative but descriptive and comparisons should not be drawn requiring statistics as it is under-powered.

We agree with the reviewer that increasing the number of subjects included in both arms of the study would be highly desirable and would significantly increase the power and impact of our study. However, due to the complexity and expense associated with scRNAseq studies, we were forced to make some pragmatic study design decisions. As mentioned above, during the design phase of this project we had to weigh including more subject in the analysis against analyzing more timepoints per subject. We made the decision to focus on the temporal aspect of DENV-elicited inflammation.

We also agree with the reviewer that it would have been highly desirable to case control and age match the subjects included in the natural infection and DENV challenge arms of the study. However, due to its endemic status in Thailand, primary DENV infections are almost exclusively observed in children, making age matching the experimental DENV-1 challenge arm of this study extremely challenging.

We have added additional language to the manuscript emphasizing these considerations.

In terms of manuscript writing quality, the introduction has a bit too much results/discussion at the end of it and these points could be saved for the discussion section and streamlined a bit in the last paragraph of the intro. A little more information on the 45AZ5 challenge strain could be included in the introduction, not just based on the citation. It would be good to add the method of attenuation and the results of the Phase 1 study (still under consideration as a vaccine candidate?) if available.

We have added additional text to the Methods and Results section of the manuscript describing the DENV challenge strain in more detail and how it was generated and selected for this study.

For the "natural" infection group, were these not also examples of "controlled" infection? Presumably these patients survived/recovered with a normal disease course? There is not an indication in the data presented that they had higher viremia either.

We apologize for the imprecise language. As the reviewer correctly states, the "natural" infection subjects did indeed "control" their infections and experienced a normal disease course.

Our use of the term "controlled" in this manuscript was intended to reflect the fact that the experimental infections in the DENV challenge arm of the study were carried out in a "controlled" setting. We have modified the language in the manuscript to eliminate this unintentional ambiguity.

Were the CD16hi monocytes the targets of infection? It is not clearly shown in the data. What percentage of the monocyte population were CD16hi monocytes? Is this the majority of the monocyte population or a subset of them?

We were unable to detect any cell-associated DENV genomic RNA in our analysis. This is despite the fact that we have published that it is possible to capture DENV genomic RNA using the 10x Genomics platform [3]. However, we have only observed this using *in vitro* infected cell lines or PBMC samples from <u>secondary</u> natural DENV infections [3]. We speculate that this could relate to the lower cell-associated DENV burden observed during primary DENV infection relative to secondary DENV infection [4], as well as the inherently inefficient nature of capturing non-polyadenylated transcripts using an oligo(dT) primer.

$CD16^{hi}$ monocytes comprised on average 20.2% (7% - 32%) of all monocytes captured in this analysis. The abundance of all annotated cell populations is included in Supplemental Table 3 and Supplemental Table 4.

This sentence from the discussion doesn't make sense how the authors are drawing causation between these two things: "Our observation that monocytes appear to respond more dramatically to DENV-elicited systemic inflammation than B cells suggests that while monocytes are more permissive to infection in vitro, they become a much less attractive target of infection and replication in vivo." Is this true that monocytes are less permissive in vivo and is it true that the responses relative to B cells give insight into this?

We apologize for the unclear language in this portion of the Discussion. Our intent was to offer a potential explanation for some of the discordant data that currently exists in terms of the primary cellular reservoir of DENV during an acute infection.

Monocytes, macrophages, dendritic cells, and other phagocytes have canonically been cited as the primary human cellular reservoir and site of replication of DENV following productive host infection [5-

8]. This is primarily based on studies utilizing *in vitro* infection techniques, wherein PBMC from healthy individuals are exposed to DENV and the resulting pattern of infection assessed after a period of conventional cell culture [9-12]. However, studies utilizing direct analysis of PBMC collected during acute DENV infection have suggested that the dominant natural circulating cellular reservoir of DENV is actually B cells [3, 4, 13, 14].

In light of the fact that monocytes appear to much more vigorously upregulate expression of anti-viral gene products than B cells in our analysis, we are hypothesizing that the soluble inflammatory/immune milieu present during acute DENV infection *in vivo* differentially impacts the transcriptional profile and infectability of PBMC, rendering monocytes inhospitable to productive infection while still permitting/enhancing the ability of B cells to support viral replication.

However, this is speculation, and we have modified the language in this section.

More information is needed on the statistical tests used in the "Statistical analysis" section of the manuscript.

We have added additional language to the Statistical Analysis section of our manuscript, capturing information that we previously only included in the single cell RNA sequencing analysis section of the Methods.

<u>Reviewer #3</u>:

These experiments are expensive and difficult to do. However, the work is very descriptive and is limited in scope in terms of patient numbers with study groups of 3 and 2, making any comparisons very limited in scope.

We agree with the reviewer that increasing the number of subjects included in both arms of the study would be highly desirable and would significantly increase the power and impact of our study. However, due to the complexity and expense associated with scRNAseq studies, we were forced to make some pragmatic study design decisions. As mentioned above, during the design phase of this project we had to weigh including more subject in the analysis against analyzing more timepoints per subject. We made the decision to focus on the temporal aspect of DENV-elicited inflammation.

Conclusions are made that natural primary DENV infection induces expression of IFNs and ISGs and that this is responsible for the suppression of mitochondrial function and cellular translation. However, from this study, this is impossible to say. There can be no causation implied from sequencing data. As the virus is known to manipulate mitochondrial function directly, it is possible that wild type DENV-1 is better able to do this resulting in the phenotypes.

We agree with the reviewer that the data presented in this manuscript is insufficient to demonstrate a causal relationship between the upregulation of interferon-induced gene products (such as IFIs and ISGs) and the suppression of mitochondrial function and protein translation. Our intent was to highlight the extensive body of literature linking these two facets of a classical cellular interferon response. We have modified our language in the manuscript to reflect this consideration.

Information on the attenuation of the experimental human DENV-1 construct is not provided in the text making it difficult to understand what the genetic background of this virus is.

We have added additional text to the Methods and Results section of the manuscript describing the DENV challenge strain in more detail and how it was generated/selected.

No information is given on the infection status of individual cells. While the sequencing platform targets polyA,

and flaviviruses are not thought to generate transcripts with polyA tails, studies by Mike Diamonds group were able to detect viral RNAs by scRNA-seq. Can the authors include or comment on this information.

We have also observed in our own studies that it is possible to capture DENV genomic RNA using the 10x Genomics platform using *in vitro* infected cell lines or samples from secondary natural DENV infections [3]. However, no cell-associated DENV transcripts were captured in either the natural infection or experimental infection samples. We speculate that this could relate to the lower cell-associated DENV burden observed during primary DENV infection relative to secondary DENV infection [4], as well as the inherently inefficient nature of capturing non-polyadenylated transcripts using an oligo(dT) primer.

We have added additional text to the manuscript highlighting our inability to capture DENV RNA in this study.

Part III – Minor Issues: Editorial and Data Presentation Modifications

<u>Reviewer #1:</u>

The manuscript reads as if different sections were written by different people. For example, the terms "DHIM", "human challenge subset" "experimental DENV-1 infection" and "experimental DENV" are used interchangeably, even in figure panels. Similarly, wild-type infection is called "natural infection" and "primary infection" interchangeably. Please settle on one consistent term for each group.

We apologize for the lack of uniformity and have modified the manuscript to utilize more consistent language throughout.

<u>Reviewer #2:</u>

The title should probably include the aspect of peripheral blood cells.

We have modified the title to include the PBMC detail

References

1. Hafemeister, C. and R. Satija, *Normalization and variance stabilization of single-cell RNA-seq data using regularized negative binomial regression.* Genome Biol, 2019. **20**(1): p. 296.
2. Puri, B., et al., *Molecular analysis of dengue virus attenuation after serial passage in primary dog kidney cells.* J Gen Virol, 1997. **78 (Pt 9)**: p. 2287-91.
3. Sanborn, M.A., et al., *Analysis of cell-associated DENV RNA by oligo(dT) primed 5' capture scRNAseq.* Sci Rep, 2020. **10**(1): p. 9047.
4. Srikiatkhachorn, A., et al., *Dengue viral RNA levels in peripheral blood mononuclear cells are associated with disease severity and preexisting dengue immune status.* PLoS One, 2012. **7**(12): p. e51335.
5. Kyle, J.L., P.R. Beatty, and E. Harris, *Dengue virus infects macrophages and dendritic cells in a mouse model of infection.* J Infect Dis, 2007. **195**(12): p. 1808-17.
6. Miller, J.L., et al., *The mannose receptor mediates dengue virus infection of macrophages.* PLoS Pathog, 2008. **4**(2): p. e17.
7. Wu, S.J., et al., *Human skin Langerhans cells are targets of dengue virus infection.* Nat Med, 2000. **6**(7): p. 816-20.
8. Schmid, M.A. and E. Harris, *Monocyte recruitment to the dermis and differentiation to dendritic cells increases the targets for dengue virus replication.* PLoS Pathog, 2014. **10**(12): p. e1004541.
9. Blackley, S., et al., *Primary human splenic macrophages, but not T or B cells, are the principal target cells for dengue virus infection in vitro.* J Virol, 2007. **81**(24): p. 13325-34.
10. Kou, Z., et al., *Monocytes, but not T or B cells, are the principal target cells for dengue virus (DV) infection among human peripheral blood mononuclear cells.* J Med Virol, 2008. **80**(1): p. 134-46.
11. Marovich, M., et al., *Human dendritic cells as targets of dengue virus infection.* J Investig Dermatol Symp Proc, 2001. **6**(3): p. 219-24.
12. Halstead, S.B., E.J. O'Rourke, and A.C. Allison, *Dengue viruses and mononuclear phagocytes. II. Identity of blood and tissue leukocytes supporting in vitro infection.* J Exp Med, 1977. **146**(1): p. 218-29.
13. Baclig, M.O., et al., *Flow cytometric analysis of dengue virus-infected cells in peripheral blood.* Southeast Asian J Trop Med Public Health, 2010. **41**(6): p. 1352-8.
14. King, A.D., et al., *B cells are the principal circulating mononuclear cells infected by dengue virus.* Southeast Asian J Trop Med Public Health, 1999. **30**(4): p. 718-28.
